# Supplementary material for: Ionic Liquids as Performance Ingredients in Space Lubricants
Source: Molecules. 2021 Feb 14;26(4):1013. doi: 10.3390/molecules26041013 (PMC7918859; doi:10.3390/molecules26041013)
Supplement: Supplementary file 1 [file molecules-26-01013-s001.pdf]

*Supplementary Material*

# Ionic Liquids as Performance Ingredients in Space Lubricants

**Erik Nyberg [1,*], Christoph Schneidhofer [2], Lucia Pisarova [2], Nicole Dörr [2] and Ichiro Minami [1]**

[1] Luleå University of Technology, Department of Engineering Sciences and Mathematics, Division of Machine Elements, SE-97187, Luleå, Sweden; ichiro.minami@ltu.se
[2] AC2T research GmbH, AT-2700, Wiener Neustadt, Austria; Christoph.Schneidhofer@ac2t.at (C.S.); Lucia.Pisarova@ac2t.at (L.P.); Nicole.Doerr@ac2t.at (N.D.)
* Correspondence: erik.nyberg@ltu.se

Academic Editors: XX
Received: 15 Jan 2021; Accepted: 11 Feb 2021; Published: XX Feb 2021

---

**Contents**

1. Material data
2. Normalized wear index
3. Numeric data for Film Parameter
4. Friction Data for $\mu_{avg}$-$W_{Hz}$ charts
5. Wear Data for $\mu_{avg}$-$W_{Hz}$ charts
6. EDS results at 300 N.

## 1. Material data

The data for the neat lubricants is listed in Table S1. Data for MAC and PFPE is taken from specifications, and P-SiSO and P-TFSA was measured using a Stabinger viscometer (SVM 3000, Anton Paar, Graz, Austria). This data is used to calculate the viscosity of the MAC & P-SiSO mixture.

Table S1. Properties of neat fluids.

| Lubricant | ν40 [mm2/s] | ν100 [mm2/s] | VI | Pour point [°C] | Comment |
|---|---|---|---|---|---|
| P-SiSO | 263 | 30 | 153 | -30 | IL |
| MAC | 108 | 14.6 | 137 | -55 | Base Fluid |
| PFPE | 157 | 49 | 358 | -60 | Reference |

The ball and disc specimen used for the SRV-2 tribology experiments conform to the standard ASTM D6425-19. Their roughness were also measured with scanning white light profilometry (SWLI), and confirmed that the composite Sq was correct to within 10 nm. Sq values for the MVT-2 samples were measured using (SWLI) and are reported in Table S2.

Table S2. Root mean square surface roughness (Sq) of steel samples.

| | Nominal Ball Sq [μm] | Nominal Disc Sq [μm] | Composite Sq [μm] |
|---|---|---|---|
| SRV-3 | 0.027 | 0.055 | 0.061 |
| MVT-2 | 0.008 | 0.005 | 0.009 |

## 2. Normalized wear index

The normalized wear index, HzD, shown in Equation S1 is described in references [1], [2].

$$\mathbf{HzD} = \left(\frac{\mathbf{3PR^*}}{\mathbf{2E^*}}\right)^{\frac{1}{3}} \quad \text{(S1)}$$

P refers to applied load, $\mathbf{R}^*$refers to the effective contact radius, and $\mathbf{E}^*$refers to the effective elastic modulus. Further details are found in [3].

## 3. Numeric data for Film Parameter

SRV-3 at 150 N:
Rx = 5e-3 m

U = 1.0749e-11
G = 2.5251e+03
W = 2.7563e-05

SRV-3 at 300N:
W = 5.5126e-05

MVT-2:
Rx = 3.175e-3 m
U = 3.2171e-12
G = 2.5251e+03
W = 1.8229e-05

k = 1.03 under all conditions.

## 4. SRV-3 friction Data for $\mu_{avg}$-$W_{Hz}$ charts

All SRV-3 friction-time plots corresponding to Figure 5 are shown in Figure S1.

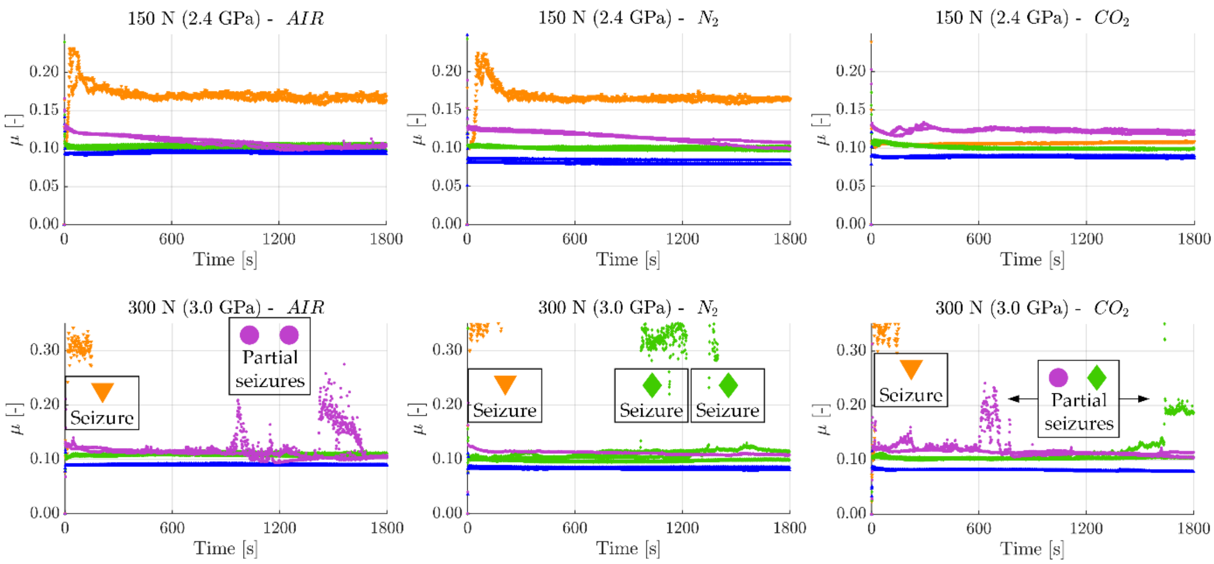


Figure S1. Friction traces recorded for neat MAC, neat PFPE, 'MAC & P-SiSO', and neat P-SiSO. Top row: 2.4 GPa contact pressure in Air, N2, and CO2 atmosphere. Bottom row: 3.0 GPa contact pressure in Air, N2, and CO2 atmosphere.

## 5. SRV-3 wear Data for $\mu_{avg}$-$W_{Hz}$ charts

SRV-3 wear scar images of balls and discs at 150N and balls 300 N are listed in Table S3, Table S4, and Table S5.

### SRV-3 wear scars 150 N

Table S3. Ball wear scars at 150 N (2.4 GPa). One representative image per test (each test was repeated at least twice).

| Lubricant | Air | N2 | CO2 |
|---|---|---|---|
| MAC | 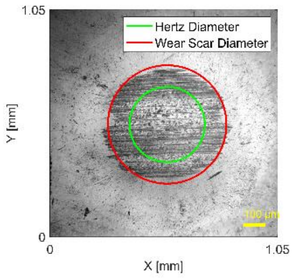 | 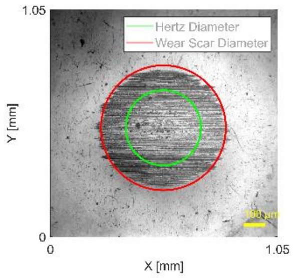 | 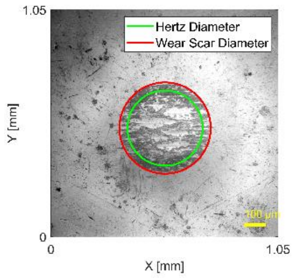 |
| MAC P-SiSO | 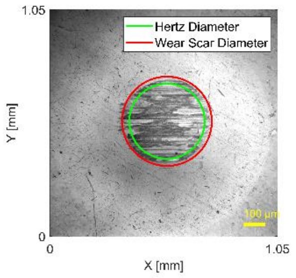 | 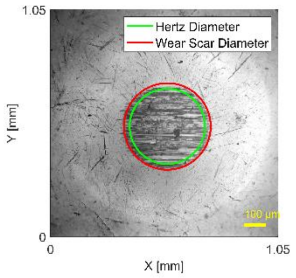 | 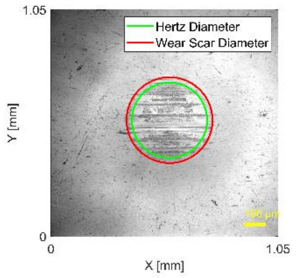 |

| | | | |
|---|---|---|---|
| P-SiSO | 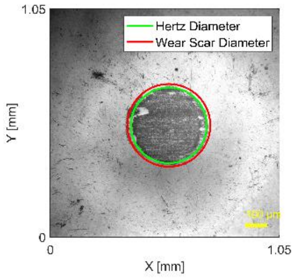1.05<br>0<br>0<br>1.05<br>Y [mm]<br>X [mm]<br>Hertz Diameter<br>Wear Scar Diameter | 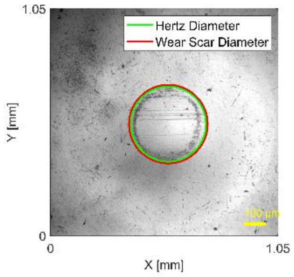1.05<br>0<br>0<br>1.05<br>Y [mm]<br>X [mm]<br>Hertz Diameter<br>Wear Scar Diameter | 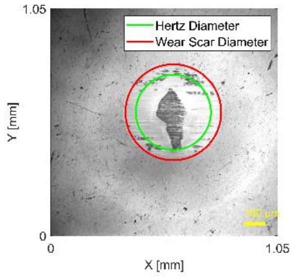1.05<br>0<br>0<br>1.05<br>Y [mm]<br>X [mm]<br>Hertz Diameter<br>Wear Scar Diameter |
| PFPE | 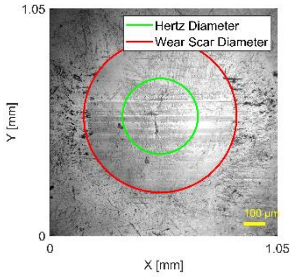1.05<br>0<br>0<br>1.05<br>Y [mm]<br>X [mm]<br>Hertz Diameter<br>Wear Scar Diameter | 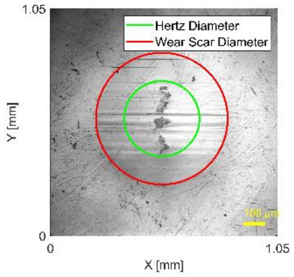1.05<br>0<br>0<br>1.05<br>Y [mm]<br>X [mm]<br>Hertz Diameter<br>Wear Scar Diameter | 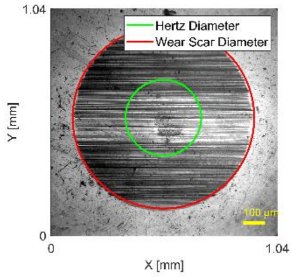1.04<br>0<br>0<br>1.04<br>Y [mm]<br>X [mm]<br>Hertz Diameter<br>Wear Scar Diameter |

Table S4. Disc wear scars from SRV-3 experiment at 150 N.

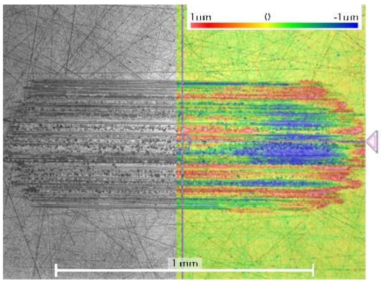

(a) MAC AIR

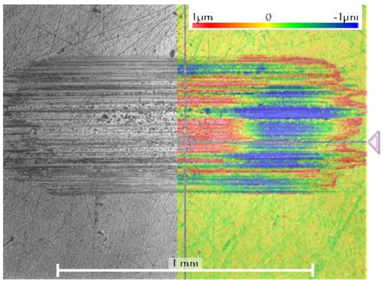

(b) MAC N2

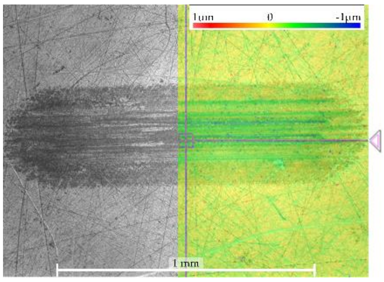

(c) MAC CO2

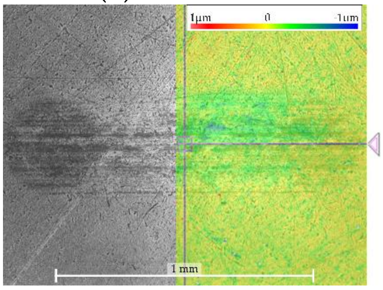

(d) MAC+P-SiSO AIR

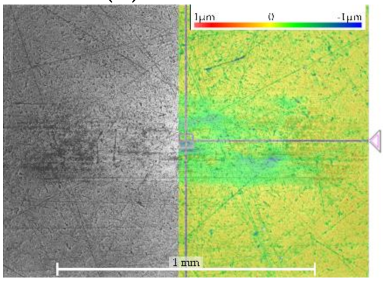

(e) MAC+P-SiSO N2

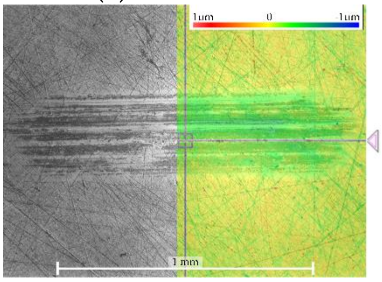

(f) MAC+P-SiSO CO2

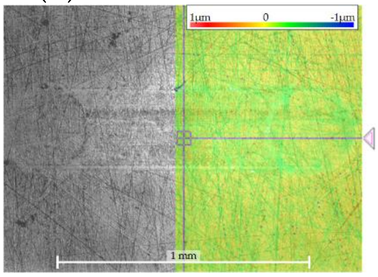

(g) P-SiSO AIR

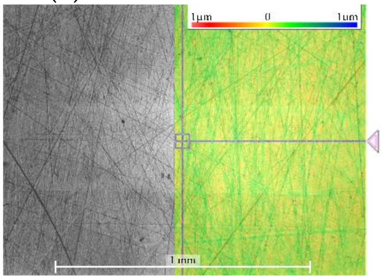

(h) P-SiSO N2

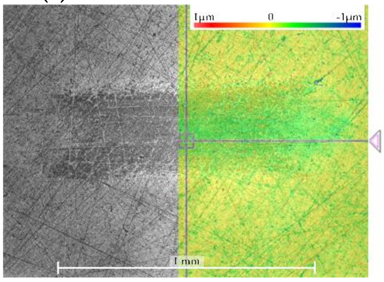

(i) P-SiSO CO2

**Wear scars 300 N**

Table S5. Ball wear scars at 300 N (3.0 GPa). One representative image per test (each test was repeated at least twice, unless the result was immediate seizure).

| Lubricant | Air | N2 | CO2 |
|---|---|---|---|
| MAC | Seizure – no data | Seizure – no data | Seizure – no data |
| MAC P-SiSO | 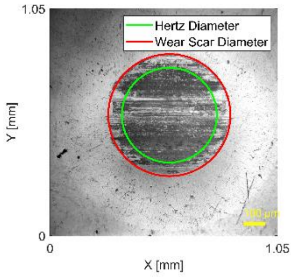  | 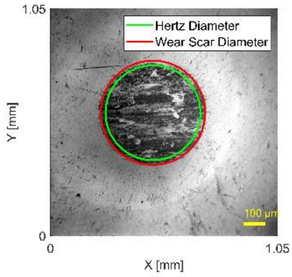  | 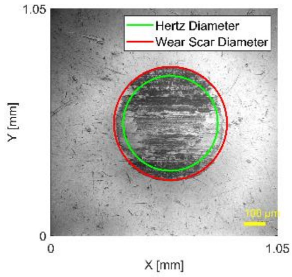  |
| P-SiSO | 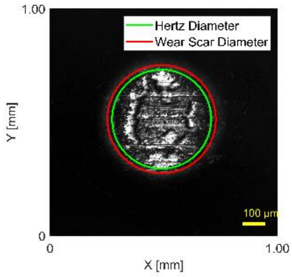  Different objective used: 2.75x2 instead of 10x0.5 | 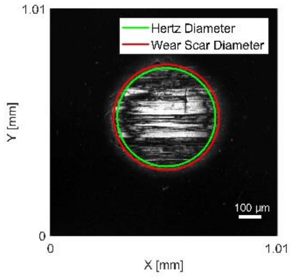  Different objective used: 2.75x2 instead of 10x0.5 | 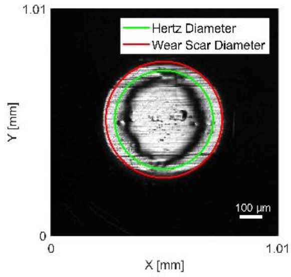  Different objective used: 2.75x2 instead of 10x0.5 |
| PFPE | 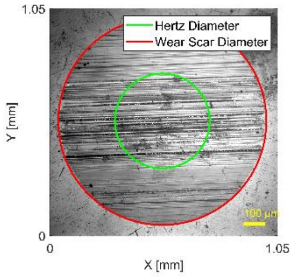  | 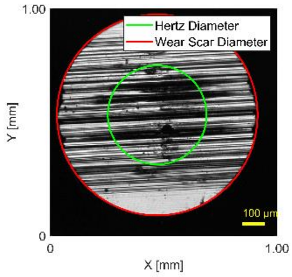  Different objective used: 2.75x2 instead of 10x0.5 | 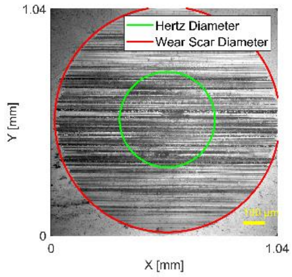  |

## 6. EDS results at 300 N

EDS results from SRV-3 {300 N / 3.0 GPa / ATM} are displayed in Figure S2.

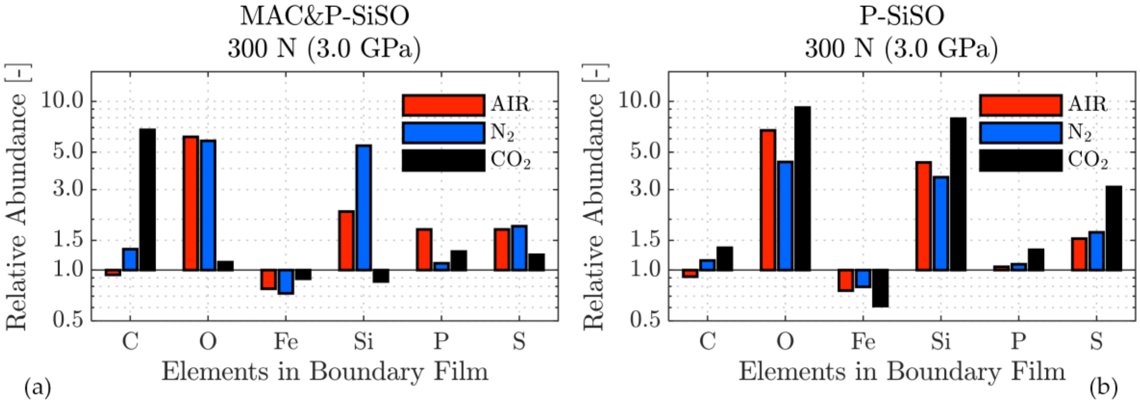


Figure S2. EDS results from SRV-3 {300 N / 3.0 GPa / ATM}.

## References


[1] M. Kalin and J. Vižintin, “Use of equations for wear volume determination in fretting experiments,” *Wear*, vol. 237, no. 1, pp. 39–48, 2000.

[2] E. Nyberg, J. Mouzon, M. Grahn, and I. Minami, “Formation of Boundary Film from Ionic Liquids Enhanced by Additives,” *Appl. Sci.*, vol. 7, no. 5, p. 433, 2017.

[3] K. L. Johnson, *Contact mechanics*. Cambridge: Cambridge Univ. Press, 1985.
